# High coverage needle/syringe programs for people who inject drugs in low and middle income countries: a systematic review

**DOI:** 10.1186/1471-2458-13-53

**Published:** 2013-01-19

**Authors:** Don C Des Jarlais, Jonathan P Feelemyer, Shilpa N Modi, Abu Abdul-Quader, Holly Hagan

**Affiliations:** 1 The Baron Edmond de Rothschild Chemical Dependency Institute, Beth Israel Medical Center, New York City, USA; 2 Centers for Disease Control and Prevention, Atlanta, USA; 3 College of Nursing, New York University, New York City, USA

**Keywords:** HIV, Hepatitis C, Needle and syringe programs, Syringe exchange programs, People who inject drugs, Injecting drug use

## Abstract

**Background:**

Persons who inject drugs (PWID) are at an elevated risk for human immunodeficiency virus (HIV) and hepatitis C virus (HCV) infection. In many high-income countries, needle and syringe exchange programs (NSP) have been associated with reductions in blood-borne infections. However, we do not have a good understanding of the effectiveness of NSP in low/middle-income and transitional-economy countries.

**Methods:**

A systematic literature review based on PRISMA guidelines was utilized to collect primary study data on coverage of NSP programs and changes in HIV and HCV infection over time among PWID in low-and middle-income and transitional countries (LMICs). Included studies reported laboratory measures of either HIV or HCV and at least 50% coverage of the local injecting population (through direct use or through secondary exchange). We also included national reports on newly reported HIV cases for countries that had national level data for PWID in conjunction with NSP scale-up and implementation.

**Results:**

Studies of 11 NSPs with high-coverage from Bangladesh, Brazil, China, Estonia, Iran, Lithuania, Taiwan, Thailand and Vietnam were included in the review. In five studies HIV prevalence decreased (range −3% to −15%) and in three studies HCV prevalence decreased (range −4.2% to −10.2%). In two studies HIV prevalence increased (range +5.6% to +14.8%). HCV incidence remained stable in one study. Of the four national reports of newly reported HIV cases, three reported decreases during NSP expansion, ranging from −30% to −93.3%, while one national report documented an increase in cases (+37.6%). Estimated incidence among new injectors decreased in three studies, with reductions ranging from −11/100 person years at risk to −16/100 person years at risk.

**Conclusions:**

While not fully consistent, the data generally support the effectiveness of NSP in reducing HIV and HCV infection in low/middle-income and transitional-economy countries. If high coverage is achieved, NSP appear to be as effective in LMICs as in high-income countries. Additional monitoring and evaluation research is needed for NSPs where reductions in HIV/HCV infection among PWID are not occurring in order to identify and correct contributing problems.

## Background

Human immunodeficiency virus (HIV) and hepatitis C (HCV) are relatively efficiently transmitted through the multi-person use (sharing) of needles and syringes used for injecting illicit psychoactive drugs [1,2]. There are an estimated 16 million people who inject drugs (PWID) in the world, of whom 13 million live in low and middle-income countries (LMIC) [3]. PWID who live in LMIC are generally at very high risk for infection with HIV and HCV. High rates of HIV prevalence (>20%) have been reported among PWID in Eastern Europe, Asia and Africa [3].

Programs to provide PWID with access to sterile needles and syringes (NSPs) are generally considered to be among the most effective means of reducing HIV and HCV transmission among PWID [4,5]. There is a considerable body of research on NSP, and syringe exchange programs in particular. There is general consensus that these programs reduce risk behavior [6] (sharing of needles and syringes) and large-scale implementation of NSP has been associated with reductions in transmission of HIV [7]. The great majority of studies of syringe exchange programs, however, have been conducted in high-income countries. For example, in a Palmateer et al. review [8], 144 of the 152 included studies were conducted in high-income countries.

There are a number of reasons why NSP may not be as effective in low/middle-income countries as in high-income countries. First, LMIC may not have the financial resources or the public health and non-governmental organizational infrastructure for successful large-scale implementation of NSP. Second, PWID in LMIC may face greater stigmatization, leading to less utilization of the programs in LMIC [9]. Third, there may be greater law enforcement interference with PWID utilizing syringe exchange programs in LMIC, particularly in locations where PWID may be incarcerated in “detention” centers or in locations where they may suffer extortion or brutality from the police [10]. Finally, NSP in high-income settings may be implemented in the context of other large scale evidence-based HIV/HCV-related prevention programs such as opiate substitution therapy/medication-assisted treatment [11]. The NSPs in high-income countries may thus benefit from the synergistic effects of “combined” prevention programming [12,13]. In contrast, NSP in LMIC are often implemented either by themselves or in the presence of other prevention programs that exist only on a pilot project scale [14].

Given the great need to reduce HIV transmission among PWID in many LMIC, and the potential barriers to the effectiveness of programs in these countries, understanding what has happened with the programs that have been implemented is likely to be critical for efforts in halting ongoing HIV epidemic among PWID.

In this review, we examine structural level NSP in different LMIC around the world to determine if implementation and scale up of NSP are associated with longitudinal changes in blood borne infection among PWID populations. By focusing on locations where NSP have been implemented and scaled compared with locations in which NSP have already been established, this review can help to elucidate the association between NSP implementation and changes in HIV/HCV prevalence and/or incidence among the PWID population served by the NSP.

## Methods

### Unit of analysis and eligibility criteria

The unit of analysis for this review was a NSP in a specific location. In our search for eligible programs, we attempted to collect as much information as possible, including published and unpublished articles, conference presentations, and “gray” literature. This differs from the usual systematic review where individual research reports or individual research studies are the standard unit of analysis. To be included in this review, the NSP had to have reached a coverage level of 50% or more of the PWID in the local population (through either direct service provision or secondary exchange, in which PWID receive syringes from NSP and distribute to their PWID peers) and provide at least 10 syringes per injector per year. This size criterion was used to distinguish between high coverage from programs that were “demonstration projects” or “pilot studies.” This level has also been shown to be an adequate coverage level to influence population level changes in rates of HIV and HCV infection [15,16]. The amount of time that was needed for each NSP to achieve structural level coverage varied; however, many of the studies achieved 50% coverage shortly after implementation, and by the follow-up biomarker measurement, all NSP had achieved structural level coverage of their PWID.

To include a specific program in the review, we located data on when the program was implemented, including when it reached the minimum size requirement, and located data on either HIV/HCV prevalence or incidence from before and after implementation of the NSP program. We required that the HIV or HCV-related data be based on laboratory testing, not on self-reported serostatus. Studies that used self-reported behavior change as their outcome measure were not included because of concerns for social desirability effects and the often imprecise association between behavior change and change in HIV/HCV infection rates [17].

A wide variety of research designs were included: randomized clinical trials, time series analyses, cohort studies, comparisons of injecting populations that received NSP with “comparable” injecting populations that did not receive NSP, and pre-post comparisons. A wide variety of research designs, including many that do not permit strong inferences about causation, were included in order to capture as many studies as possible. Programs for which we were able to locate data only after full NSP implementation were not included in the review.

Finally, the studies included had to come from locations defined by the World Bank as low/middle-income countries or transitional-economy countries [18]. Studies from high-income locations were screened by researchers, but not included in this review.

### Search methodology

**Figure 1:**
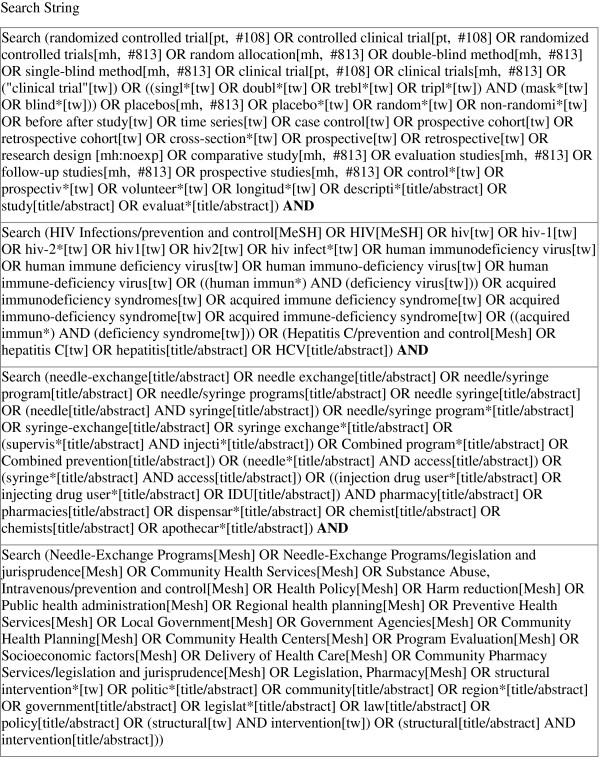
**Search terms and terminology used for retrieval of eligible citations and reports.** This figure displays the terminology used to search for relevant studies. Keyword and subject terms were retrieved from previous research of systematic reviews on similar HIV/HCV topics among PWID.

Selection of studies for this review was based on a structured literature review and analysis utilizing Preferred Reporting items for Systematic Reviews and Meta-Analysis (PRISMA) guidelines [19,20]. Systematic literature searches were conducted to identify potentially eligible articles from journals and government/country reports. Figure 1 describes in detail the search terms that were utilized for this review; the same search terminology was used for each of the databases searched, which included PubMed, EMBASE, Web of Science, and NLM Gateway. In addition, we also searched conference abstracts from International AIDS Conferences (IAC) from 2000 through 2011 and International Harm Reduction conferences (IHRA) from 2000 through 2011 [21], along with published reviews of needle and syringe exchange programs throughout the world. For locations in which HIV or HCV information was available but coverage information was not consistent (such as in Bangladesh and Vietnam), we contacted researchers via email with knowledge of the programs in each location to obtain the necessary coverage information if available, including the number of PWID that visit the NSP, the number of needles/syringes distributed, and the number of PWID that are covered by the NSP. The search included all studies published between January 1^st^ 1980 and November 30^th^ 2011.

### Data abstraction and coding

A standardized coding form was developed to document pertinent information for each program. Information collected included demographics of the injecting population covered under the NSP, characteristics of the NSP including coverage and distribution information, and information related to changes in HIV or HCV infections over time. If analysis of HIV or HCV infection data was conducted on specific subgroups (such as by age, race, gender, etc.) these data were collected as well. In addition, we reported measures of changes in subgroups or from multivariate modeling if studies reported effects with adjustment for participant characteristics in relation to changes in HIV or HCV infection information over time.

Studies were independently coded by two research associates (JF and SM). All coding forms were reviewed for quality by the co-principal investigator (HH) before finalization, with disagreements in coding reviewed by the principal investigator (DDJ).

In locations where multiple studies described the same NSP intervention, we compiled data across studies, focusing on the most complete measures of exposure to the NSP intervention with the most comprehensive review of HIV/HCV information including effect modifiers, adjustment for confounders, and length of the evaluation. We also combined data from multiple studies of the same intervention if different aspects of the data we needed were in different reports, e.g., number of PWID in one report and numbers of syringes exchanged in a different report.

### Types of interventions

We were prepared to include both syringe exchange programs and increased pharmacy sales programs as NSPs. However, all of the programs for which we were able to obtain the necessary data (see inclusion criteria above) were needle and syringe exchange programs.

### Types of outcome measures

The primary outcomes of interest in this review include changes in prevalence or incidence of HIV, HCV, HIV/HCV co-infection or changes over time in newly reported HIV or HCV cases of infection. For studies that reported these measures for a specific subgroup, such as new injectors vs. older injectors, race, age, ethnicity, location, or length of injecting career, these data were recorded as well. For studies that included multivariate modeling, adjusted effect sizes were presented if they were based on changes in HIV or HCV infection data among PWID over time. Several studies used HIV prevalence among new injectors to estimate HIV incidence among new injectors, and we also included those data.

### Analysis of included studies

We performed a systematic review of the effects of exposure to NSP on incidence or prevalence of HIV and HCV infections among PWID in selected locations. A quantitative synthesis was not conducted due to the considerable variations in time period of analyses, measures of effect size, non-comparable study designs, the specific operating characteristics of the programs, and uncertain bias across and between studies due to lack of randomization. A narrative analysis permits reviewing of selected issues, cases, or events in depth identified through the systematic review [22], allowing for a more complete and detailed analyses not constrained by predetermined categories.

## Results

### Description of search results

**Figure 2:**
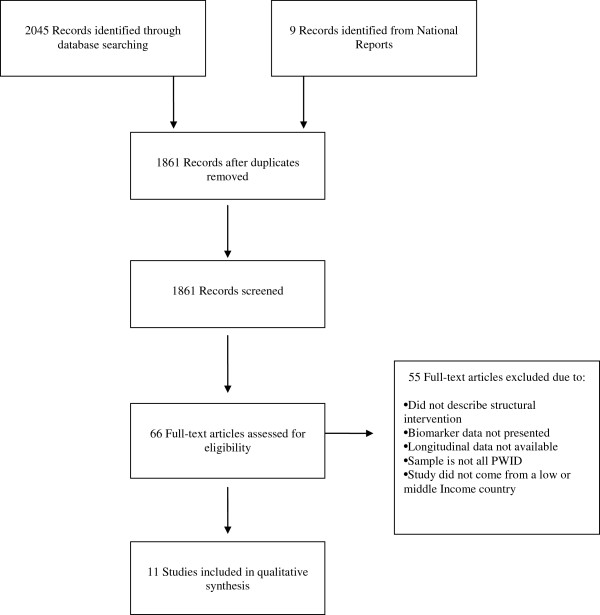
**PRISMA diagram of eligible studies included in review.** This figure displays the methodology of review of literature from databases included in the review. The PRISMA diagram outlines how the research team arrived at the eligible study list utilized in this review.

Figure 2 displays the systematic search of the literature. Four domains were included in the search terms, including NSP terminology, pharmacy terminology, biomarker terminology (HIV, HCV), and finally study design terminology. The original search comprised 1,291 citations from a systematic search reviewed by two reviewers independently (JF and SM) after removal of duplicate records not identified through the systematic importation of eligible studies from each database (NLM Gateway, PubMed, Web of Science, and EMBASE). Additionally, we performed a secondary search of needle and syringe exchange program papers utilizing reference lists from systematic review of NSP programs [8,23,24] and through collaboration with experts in the field of injection drug use in low/middle and transitional income locations. An additional 570 articles/reports were obtained and reviewed, and we identified an additional nine studies from this secondary search after removal of duplicates from the secondary search. A total of 66 full text articles were examined, and 11 qualitative studies and reports were obtained from this full text search.

### Results of the search

A total of 11 studies/reports examining nine low/middle-income or transitional-economy countries were eligible for inclusion in this NSP review based on laboratory HIV or HCV results and greater than 50% coverage of the NSP within the respective community. These 11 studies described 12 distinct locations involving NSP evaluated from 1990 through 2010. If multiple locations in a single country were examined the data are presented by location and by time period. Studies included in this review cover locations in Bangladesh, Brazil, China, Estonia, Iran, Lithuania, Taiwan, Thailand, and Vietnam.

### Review of studies, summary of results

There were six studies that documented population level changes in HIV prevalence over time and three studies that documented population level changes in HCV infection prevalence or incidence over time. Of the six studies reporting on HIV prevalence, four studies reported population level decreases in prevalence ranging from −3% to −15% while two studies reported population level increases ranging from +6% to +16%. Of the three studies reporting on population level HCV infection prevalence or incidence, all reported decreases in prevalence ranging from −4.2% to −10.1%, while one study reported a stable population level incidence of 1.2-1.3/100 person years (PY). Estimated HIV incidence among new PWID (defined as less than three years of injecting history) decreased in three studies ranging from −16/100 PY to −11/100PY. Table 1 summarizes the HIV and HCV infection prevalence/incidence studies included in the review.

**Table 1:** Summary of primary studies with HIV/HCV biomarkers

**Study Information**			**Coverage**	**Pre-Implementation and Expansion**						**Post-Implementation and Expansion**					
**Investigator**	**Location**	**Study Design**	**Syringes per PWID/Year**^**1**^	**Year**	**(n)**	**HIV prev.**	**HCV prev.**	**Estimated HIVinc.**^**2**^	**HCV inc.**	**Year**	**(n)**	**HIV prev.**^**3**^	**HCV prev.**^**3**^	**Estimated HIVinc.**^**2**^	**HCV inc.**
Gray 1998 [25]	Chiang Rai, Thailand	Time Series Cross Sectional	150-160	1993	46	33%				1996	132	18% (15%)			
Caiaffa 2003 [26]	Porto Alegre, Brazil	Pre-Post Study Comparison	6-20	1998	137	49%				2001	255	64.3% (+16%)			
Wu 2007 [27]	Dagou, China	Pre-Post Study Comparison	290-300	2002	235	40%	99%			2003	226	34% (6%)	89% (10%)		
	Luzhai, China	Pre-Post Study Comparison	140-150	2002	194	56%	89%			2003	219	53% (3%)	85% (4%)		
Azim 2008 [28]	Dhaka, Bangladesh	Time Series Cross Sectional	285-344	1990	418	2%	67%			2006	1092	7% (+5%)	57% (10%)		
Azim 2009 [29]	Dhaka, Bangladesh	Time Series Cross Sectional	285-344	1999	418				1.2/100 PY	2007	1045				1.3/100 PY
Uuskula 2011 [30]	Tallinn, Estonia	Time Series Cross Sectional	23-78	2005	350	54%		20.9/100 PY		2009	327	50% (4%)		9/100 PY	
Hammett 2012 [31]	Ning Ming, China	Time Series Cross Sectional	20-30	2002	290	17%		12/100 PY		2008	187	11% (6%)		11/100 PY	
	Lang Son, Vietnam	Time Series Cross Sectional	20-30	2002	342	46%		22/100 PY		2009	185	23% (23%)		3/100 PY	

1. Syringes per PWID/Year are given as a range based on the first and last date of data collection in each primary study location.

2. Among new PWID (persons who had begun injecting in the previous 3 years, assumed to be HIV negative when they began injecting).

3. Post Implementation columns include follow-up biomarker information in addition to the difference from baseline to follow-up.

National surveillance reports were evaluated for changes in newly reported HIV cases during NSP expansion in Iran, Taiwan, Lithuania and Vietnam. Reports from Iran, Taiwan and Vietnam reported decreases in newly reported HIV cases, ranging from −30% to −93.3%. Lithuania reported an increase of +37.6% in newly reported HIV cases among PWID. Table 2 summarizes the changes in newly reported HIV cases among PWID during implementation of NSP services.

**Table 2:** Summary of National Surveillance Data of HIV Biomarkers

**Country Report Information**			**Coverage**	**Pre Implementation and Expansion**		**Post Implementation and Expansion**	
**Investigators**	**Location**	**Surveillance Method**	**Syringes per PWID/Year**^**1**^	**Year**	**Baseline HIV cases (n)**	**Year**	**Follow-up**^**2**^**HIV cases (n) (Percent Change)**
CDC 2007 [32]	Iran	National Surveillance Data	4-41	2003	2332	2007	426 (82%)
Astrauskiene 2010 [33]	Lithuania	National Surveillance Data	56-98	2005	85	2009	117 (+38%)
CDC 2010 [34]	Taiwan	National Surveillance Data	7-67	2006	1693	2010	114 (93%)
Hammett 2010 [35]	Vietnam	National Surveillance Data	9-52	2005	11358	2009	7947 (30%)

1. Syringes per PWID/Year are given as a range based on the first and last date of data collection in respective study.

2. Post Implementation columns include follow-up biomarker information in addition to the difference from baseline to follow-up.

### Included studies

There were nine primary research articles included in this review: Azim 2008 [28], Azim 2009 [29], Caiaffa 2003 [26], Wu 2007 [27], Hammett 2006 [36], Des Jarlais 2007 [37] and Hammett 2012 [31], Uuskula 2011 [30], and Gray 1998 [25]. Additionally, there were four national surveillance reports included: Iranian MOH 2007 [32], Astrauskiene 2010 (Lithuania) [33], CDC 2010 (Taiwan) [34], and Hammett 2012 (Vietnam) [35].

### Primary study interventions

#### Dhaka Bangladesh

In Bangladesh, NSP were expanded significantly starting in 1998 with full implementation occurring in 2000. By 1999, the NSP served a population of 3,500 PWID, covered 89-93% of the PWID in Dhaka, and distributed up to three syringes and six needles per injector per week with an average needle and syringe exchange rate of 73% among program participants [28].

Azim 2008 [28] and Azim 2009 [29] recruited male PWID from NSP, detoxification centers, and clinics in Dhaka, Bangladesh to analyze prevalence and incidence of HIV and HCV infection. Azim 2008 [28] examined 3,759 male PWID from 1999–2006 and documented an HIV prevalence increase from 1.4% (1999–2000) to 7% (2006). HCV infection prevalence decreased from 66.5% (1999–2000) to 56.4% (2006).

Azim 2009 [29] analyzed PWID HIV incidence from 1998–2007. The HIV incidence among PWID remained stable at 1.16/100 PY (1998–1999) to 1.29/100 PY (2007) while HCV infection incidence among PWID decreased from 37.50/100 PY (1998–1999) to 11.58/100 PY (2007).

Azim 2009 reported decreases in several injection related behaviors; borrowing of needles/syringes decreased 10.6% while lending of needles/syringes decreased 29.8%. Additionally, long term PWID were more likely to utilize the NSP; those who had at least a 10 year history of injection drug use were nearly three times more likely to utilize the NSP compared to PWID who had been injecting for less than 10 years.

#### Porto Alegre Brazil

NSP programs in Porto Alegre were started in 1996 with cooperation from the public health system in conjunction with municipal and state authorities. In 1998, the NSP program distributed approximately 48,000 needles/syringes, increasing to over 150,000 in 2002 with an exchange rate of nearly 50%; the NSP serve a population of approximately 7,000 to 8,000 PWID.

Caiaffa 2003 [26] recruited PWID from non-institutionalized locations in Porto Alegre in 1998 (when NSP was still in its scale up and expansion period), and again in 2000–2001 (when NSP had become fully established in the city). PWID in both samples were predominately young (average age: 28–31 years old), male (81-84%), and injected mainly cocaine (74-87%). Both samples had moderate to high levels of sharing in the last six months (36-59%), and 60% had ever visited the NSP at least once. HIV prevalence among PWID in Porto Alegre during NSP implementation increased from 48.5% (1998) to 64.3% (2000–2001). No explanation was given by authors related to the changes in HIV infection prevalence seen among locations without NSP in place.

#### Dagou & Luzhai China

In 1998 the Ministry of Health of China promoted social marketing of safe injection as part of their HIV/AIDS prevention strategy. The first NSP were started in Dagou and Luzhai with ramp up beginning in 2002. The number of NSP locations in China increased from 93 sites in March 2006 to 729 by December 2006.

Wu 2007 [27] analyzed the impact of NSP on HIV prevalence in Dagou and Luzhai and compared results to two cities in China that did not have NSP programs in place, Yu’nan and Yongning. PWID were recruited into the study from several locations including detoxification centers and community outreach. In all four locations in which PWID were recruited (Dagou, Luzhai, Yu’nan, and Yongning), PWID were predominantly male and between 20 and 39 years of age.

Both locations that had NSP documented decreases in HIV and HCV infection prevalence. In Dagou, HIV prevalence decreased from 40% (2002) to 33.6% (2003) (p = 0.16), while HCV infection prevalence decreased from 98.7% (2002) to 88.5% (2003) (p < 0.01). In Luzhai, HIV prevalence decreased from 56.2% (2002) to 53.2% (2003) (p = 0.54), while HCV infection prevalence decreased from 88.7% (2002) to 84.5% (2003) (p = 0.22).

In the cities that did not have NSP in place, changes in HIV and HCV infection prevalence varied. In Yu’nan, HIV prevalence remained stable at 17.6% (2002–2003) (p = 0.99), while HCV prevalence decreased from 88.1% (2002) to 58.5% (2003) (p < 0.01). In Yongning HIV prevalence increased from 22.4% (2002) to 24.1% (2004) (p = 0.68) while HCV infection prevalence increased from 81.6% (2002) to 88.2% (2004) (p = 0.07). No explanation was given by authors related to the changes seen in HIV and HCV infection prevalence seen among locations without NSP in place.

#### Lang Son Province Vietnam & Ning Ming County China

The “Cross-Border” intervention took place in the regions of Lang Son Province, Vietnam and Ning Ming County in the Guangxi province in China. The intervention involved packaged harm reduction services, including a pharmacy-based voucher program for acquiring clean needles/syringes along with clean injecting equipment and condoms. On average, 7,000 to 10,000 needles/syringes were distributed per month at each location, serving a population of approximately 3,000 PWID in each region. Three studies reported on changes in HIV prevalence in these locations, Hammett 2006 [36], Des Jarlais 2007 [37] and Hammett 2012 [31]; this review includes the more complete analysis utilizing a longer follow-up period from Hammett 2012.

Hammett 2012 [31] documented changes in HIV prevalence and HIV incidence among new PWID over an eight year period in conjunction with NSP expansion in both locations beginning in 2002 of which six years of data was available for Ning Ming while seven years of data was available for Lang Son. 2125 PWID were included in the Ning Ming sample and 2677 PWID were included in the Lang Son sample.

In Ning Ming, HIV prevalence decreased from 17% to 14% after 12 months, and then stabilized at 11% after 72 months (p = 0.003). In Lang Son, HIV prevalence decreased to 43% after 12 months and decreased further to 23% after 84 months (p < 0.001). When examining only new PWID, defined as injectors that had injected for three years or less, HIV incidence in Ning Ming decreased from 12/100 PY to 9/100 PY after 12 months and stabilized at 11/100 PY after 72 months. Among new PWID in Lang Son the HIV incidence decreased from 22/100 PY to 16/100 PY after 12 months and decreased further to 3/100 PY after 84 months (p < 0.001).

#### Tallinn Estonia

NSP was implemented in Estonia in 2003 with the majority of services provided in the capital city of Tallinn, where nearly 75% of the PWID in Estonia are located. The NSP serve a population of approximately 10,000 PWID and have increased needle/syringe distribution greatly since implementation, from 18,000 needles/syringes distributed in 2003 to over 770,000 by 2009.

Uusküla 2011 [30] recruited 1,027 PWID between 2005 and 2009, of which 168 were new injectors (defined as having injecting histories of three years or less). The sample was predominately male (80%), Russian (80%), and young (mean ages: 24 to 27 years old). Among new injectors, subjects were predominately male (74-82%), Russian ethnicity (78-89%), and mainly injected fentanyl (48-61%) or amphetamine (32-47%). High levels of receptive needle/syringe sharing were documented among new PWID, with rates ranging from 74-79%.

HIV prevalence in the entire sample decreased slightly from 54% (2005) to 50% (2009). However, when examining only new injectors, HIV prevalence decreased from 34.2% (2005) to 15.8% (2009) (p = 0.046); after controlling for age, gender, injection frequency and NSP use, the change in overall HIV prevalence among new injectors remained statistically significant (*χ*2 = 8.31, p = 0.016). Estimated HIV incidence among new injectors decreased from 20.9/100 PY (2005) to 9/100 PY (2009) (p = 0.026).

Several injection-related behaviors were measured in the study among the PWID sample; during the study period, receptive sharing of needles/syringes decreased 5% and the percentage of injectors that utilized NSP increased from 44% in 2005 to 76% in 2009.

#### Chiang Rai Thailand

The NSP in Chiang Rai Province Thailand originally began in 1992 in three of the nine Akha hill tribe villages in northern Thailand. Five thousand needle and syringe kits were provided by the government for vaccination and were subsequently distributed among 46 PWID in three villages from 1992 to May 1994. During the period of evaluation, needles were not allowed to be distributed for the purposes of needle exchange; however, there was an agreement made that allowed for this small village to receive needles and syringes in response to the elevated number of new infections among PWID in the hill tribe villages. In 1995 a grant from the Australian government allowed implementation of NSP in all nine villages. The NSP served approximately 132 PWID and allowed up to 12 needles/syringes to be acquired per month for each PWID.

Gray 1998 [25] analyzed the impact of the NSP on HIV prevalence among PWID in Akha hill tribe villages in Chiang Rai Province Thailand from 1993–1996. All PWID in this location were included in the study; 46 were part of the 1993–1994 sample while 132 were part of the follow-up 1995–96 sample. The samples were typically male (85%) and injected primarily heroin.

Over the course of the study, the HIV prevalence among PWID decreased from 33% (1993) to 18% (1995–1996).

### National surveillance report interventions

#### Islamic Republic of Iran

The first NSP in the Islamic Republic of Iran was established in 2003 [32]. Programs slowly increased after 2003 as laws were reformed so PWID were not arrested if they were covered by prevention and care services including drop-in centers which offer NSP [38]. In 2007 there were 4,665,512 needles/syringes distributed, with an average of 41 needles/syringes distributed per PWID per year; during the same time period NSP sites increased from 170 in 2008 to 637 by 2010 [21].

The Center for Disease Management at the Iranian Ministry of Health reported national surveillance data on annual newly reported cases of HIV among PWID undergoing testing at surveillance sites from 1986 to 2007. The number of newly reported cases among PWID continued to increase through the late 1990s and early 2000s with 2332 cases in 2003, and a peak of 3145 new cases in 2004. However, with the implementation and scale-up of NSP services, the number of newly reported cases among PWID began to drop to 2,293 cases in 2005, 1,658 cases in 2006, and 426 cases in 2007.

#### Lithuania

In early 1997 NSP was first introduced in Lithuania via mobile and illegal underground needle/syringe distribution. In 2006 NSP was legalized in Lithuania, and by 2009, there were 12 NSP sites in Lithuania serving approximately 3,200 PWID. The number of needles/syringes distributed increased starting in 2006 and by 2008 313,000 needle/syringes were distributed to PWID, decreasing to 188,000 in 2009. No explanation was given by the report as to the reason for the decrease in distribution in the last year of data analysis.

Astrauskiene 2010 [33] reported national newly reported HIV cases among PWID, collected by the Ministry of Health of Lithuania. The number of newly reported HIV infections among PWID decreased from 85 cases in 2005 to 62 cases in 2006, 59 cases in 2007, and 42 cases in 2008. However, in 2009, there was an increase with 117 newly reported cases among PWID. The increase in cases in 2009 coincided with a significant decrease (40%) in needle/syringe distribution.

#### Taiwan

In 2005, Taiwan instituted NSP on a trial basis throughout the country. By 2006 NSP in Taiwan had ramped up to full-scale operation with a 900% increase in coverage of PWID during the first two years of operation. A total of 450,000 needles/syringes were distributed in the first year, expanding to nearly four million in 2007, serving approximately 60,000 PWID.

A 2010 Taiwan Center for Disease Control (CDC) [34] document reported national surveillance data of newly reported HIV cases among PWID. Surveillance data captured in this analysis include all HIV incident PWID cases registered with CDC Taiwan between 2006 and 2010. The number of newly reported HIV cases among PWID decreased from 1,693 cases in 2006 to 114 cases in 2010.

#### Vietnam

NSP programs in Vietnam were started as pilot projects in 1993, with major scale-up occurring in 2005 as a result of the HIV Prevention Project implemented by the World Bank. In 2006, HIV/AIDS law mandated harm reduction activities that included NSP, leading to expansion from 21 provinces in 2005 to over 60 provinces by 2009. During this same time period, the number of needles/syringes distributed increased from two million in 2006 to over 11 million in 2007, serving approximately 215,000 PWID.

Vietnam surveillance data among at risk groups collected newly reported HIV cases from 2005 through 2009, coinciding with the period of expanding NSP in the country [35]. During this time period, the number of HIV cases among PWID decreased from 11,358 in 2005 to 7,947 cases in 2009.

## Discussion

Before considering the substantive findings, it will be useful to consider limitations on the quantity and quality of the data on NSPs in low-and middle-income countries. As noted in the introduction, previous systematic reviews of NSPs in LMICs included only two programs, while we were able to locate data from 11 programs, despite stricter eligibility criteria such as biological measures of HIV or HCV as outcomes. Still, that we were able to obtain the needed information for only a modest number of NSP in LMICs raises concerns. Given our difficulties in finding data on NSPs in LMICs, we have to conclude that that the NSPs included in our systematic review probably do not reflect typical operations of NSPs in low/middle-income countries. As programs with better implementation are probably more likely to report data, it is likely that the data included in our review probably reflect NSPs with better reporting mechanisms, supportive policies, or program operations than “average.”

The data for the programs included in this study often had considerable imprecision and variability. Coverage in terms of the percentage of PWID who were reached or the numbers of syringes exchanged per PWID per year was often quite imprecise, as the estimates of PWID in the local area often had very wide ranges, and the numbers of syringes exchanged was sometimes reported as a range rather than a simple count.

Another aspect of imprecision in the data comes from using changes in HIV and/or HCV infection prevalence and newly reported HIV cases among PWID as outcome measures. Changes in HIV or HCV infection incidence would be the most desirable outcome measure, but are quite difficult to measure for both practical and ethical reasons. An ethically conducted cohort study would need to provide good access to sterile injection equipment to subjects and thus may not reflect HIV or HCV infection incidence in the local PWID population. Estimating HIV incidence from HIV prevalence among new injectors requires the assumption new injectors were HIV seronegative when they began injecting, which is may not be valid if there is substantial sexual transmission in the local area. Changes in HIV/HCV co-infection prevalence reflect both changes in the rate of new infections and differential loss of HIV/HCV-infected versus HIV/HCV-uninfected persons in the local population. (It is likely that more HIV seropositives will be lost from the active injecting population than HIV-negatives due to HIV-related morbidity and mortality). Changes in the numbers of newly-diagnosed HIV infections among PWID also have limitations as an outcome measures. They may reflect changes in surveillance methods and even if the surveillance methods do not change, the time between when a new HIV infection occurs and when it is reported may be substantial and the reporting lag may itself change over time. We did not formally grade the quality of the data in the different studies, as there typically was not sufficient detail reported in the studies for differentiating data quality among these studies. It is our impression, however, that there was no relationship between the quality of the data and the size of the decline in HIV or HCV prevalence across the studies.

Additionally, most of the changes in HIV/HCV infection incidence or prevalence in the studies reported here also suffer from the lack of any comparison group.

All of these methodological limitations are likely to persist in assessing the effectiveness of large-scale NSP for HIV prevention among PWID. Communities are the appropriate unit of analysis for assessing implementation of high coverage NSP programs, so any studies with comparison groups would have to be very large and would likely be logistically complex. Also, given the current data on the effectiveness of NSPs, it would be unethical to fail to provide NSPs to PWID simply for the sake of conducting research.

### Interpretation

Despite the limited quantity of data on NSPs in low-and middle-income countries, we do believe that the data presented here can be used to address three critical questions about NSPs in LMICs.

1. Is there a “critical coverage” level of syringe distribution and PWID in NSP to affect change in HIV or HCV infection incidence or prevalence? The numbers of syringes distributed per PWID per year varied greatly among the studies reported here, and there are only a modest number of studies. Nevertheless, the data presented in Table 1 suggest that 20–30 syringes per year per PWID might be considered a minimum level of coverage that would lead to population-level effects among opiate-using populations [16]. Note that in the Porto Alegre study, only 20 syringes were distributed per PWID per year, and cocaine was the primary drug injected. As cocaine is often injected at very high frequencies, it is likely that more syringes per PWID per year are needed to control HIV transmission among cocaine-injecting populations.

2. What is the “causal lag” time period before a high coverage NSP will lead to changes in HIV incidence or prevalence in a population of PWID? With the exception of the Bangladesh studies, all of the studies reported here showed the changes in HIV or HCV infection incidence or prevalence occurring within relatively short time periods, typically 2 to 4 years. This does suggest that implementation of high coverage of PWID and sterile injection equipment may have effects within very short time periods [39].

3. Is high-coverage of sterile injection equipment and PWID in NSP programs in LIMCs as effective as NSPs in high-income countries? A number of the studies reported here do show rather substantial reductions in HIV infection associated with the implementation of high coverage NSPs. These are most clearly seen in the studies that measured HIV incidence or estimated HIV incidence (through prevalence among new injectors) or used newly reported HIV cases. In the Estonia study, estimated HIV incidence among new injectors fell by half while in the Cross-Border studies (China and Vietnam), estimated HIV incidence among new injectors fell by approximately three quarters or more. Even in the Bangladesh study, where HIV incidence was stable over eight years at slightly over 1/100 person-years at risk, keeping incidence this low over such an extended period can be considered a prevention success.

For the NSP where newly reported HIV cases were used as an outcome measure, the reductions ranged from 30% to 93%. The data from Lithuania, however, showed a substantial increase in newly reported HIV cases in the last year of the study period, coincident with a reduction in syringes distributed during that year. There also has been a recent outbreak of HIV among PWID in Greece that was associated with low implementation of prevention services [40,41].

Overall, the data in Table 1 suggests that NSPs in LMIC show similar rates of reduction in HIV incidence and prevalence as NSPs in high-income countries [8]. There are, however, examples from both high-income and LMICs where NSPs have not prevented outbreaks of HIV transmission among PWID [39,42].

With the possible exceptions of the estimated HIV prevalence in Guangxi province China [27] and the numbers of newly reported HIV cases in Taiwan, the studies reviewed here provide little evidence that the NSPs in these locations are eliminating injecting-related HIV transmission in the local population of PWID. Some of the estimated incidence rates are still unacceptably high, from 4/100 to 9/100 person-years at risk. Large-scale “combined” HIV prevention programming, with not only NSPs but also substance dependence treatment, HIV counseling and testing and anti-retroviral treatment for HIV seropositives may be required to reduce HIV transmission to a level where zero new injecting-related infections becomes a realistic public health goal.

## Conclusions

We conducted a systematic review of NSPs in low-and middle-income countries. The inclusion criteria included high coverage of at least 50% of local PWID participating in the program or a minimum of 10 syringes distributed per PWID per year, HIV and/or HCV biomarker outcome data, and data for pre and post high-coverage implementation of the NSP. We were able to locate the needed data for only 11 such NSPs. (Previous reviews had included only two of these NSPs). The data suggest that a minimum of 20 to 30 syringes per PWID per year may be needed to affect HIV and/or HCV transmission in a population of PWID. Reaching high coverage levels (between 20–30 syringes per PWID per year and at least 50% of the PWID population) for NSP is very likely to be followed by reductions in HIV and/or HCV infection in the local population of PWID [16]. Additional prevention and treatment services for PWID may be needed, however, in order to eliminate HIV among PWID in low and middle income countries.

## Competing interests

The authors declare that they have no competing interests.

## Authors’ contributions

JF and SM conducted the review of the literature, extracted the pertinent data, and performed analysis of the individual study and national surveillance data; DDJ, JPF and HH wrote the first draft of the manuscript; HH and DDJ provided strategic advice and assisted with editing of the manuscript; DDJ supervised the project, and all authors read and approved the final manuscript.

The findings and conclusions in this report are those of the authors and do not necessarily represent the views of the organizations and/or agencies the authors are affiliated with.

## Pre-publication history

The pre-publication history for this paper can be accessed here:

http://www.biomedcentral.com/1471-2458/13/53/prepub
